# Rehabilitation Therapy Utilization in Patients with Parkinson's Disease in Korea

**DOI:** 10.1155/2018/9475415

**Published:** 2018-11-14

**Authors:** Han Gil Seo, Sang Jun Park, Jiah Seo, Seong Jun Byun, Byung-Mo Oh

**Affiliations:** ^1^Department of Rehabilitation Medicine, Seoul National University College of Medicine, Seoul National University Hospital, Seoul, Republic of Korea; ^2^Department of Ophthalmology, Seoul National University College of Medicine, Seoul National University Bundang Hospital, Seongnam, Republic of Korea; ^3^Big Data Center, Seoul National University Bundang Hospital, Seongnam, Republic of Korea

## Abstract

**Objective:**

Although evidence and guidelines recommend appropriate rehabilitation from the beginning of diagnosis in patients with Parkinson's disease (PD), there is a lack of data addressing the utilization of rehabilitation therapies for these patients in practice. The aim of this study is to investigate the rate of rehabilitation therapy utilization over time in patients with PD using a nationwide cohort in Korea.

**Methods:**

Patients were identified using the registration code for PD in the program for rare, intractable disease from the National Health Insurance Service-National Sample Cohort database, which consists of 979,390 Korean residents. Data were divided into four periods: 2004–2006, 2007–2009, 2010–2012, and 2013–2015. We assessed the utilization of rehabilitation therapies and the associated patient characteristics.

**Results:**

The numbers of patients with PD were 384 in 2004, 855 in 2007, 1,023 in 2010, and 1,222 in 2013. The numbers of physiatrist visits per person were 0.58, 0.96, 1.97, and 2.91, in the respective periods. Among the patients, 35–40% had claims for physical therapy, 16–19% for occupational therapy, and 4–6% for swallowing therapy. There were no remarkable differences between these rates between the study periods. Sex, age, income, disability, and levodopa-equivalent dose were significantly associated with the utilization of rehabilitation therapy.

**Conclusion:**

This study demonstrated that the rate of rehabilitation therapy utilization did not change remarkably in patients with PD from 2004 to 2015 in Korea although the number of physiatrist visits increased dramatically. The present evidence and guidelines may have not been adequately integrated into clinical practice during the period of study. Additional efforts may be warranted to provide adequate rehabilitation therapies in clinical practice for patients with PD.

## 1. Introduction

Parkinson's disease (PD) is a progressive neurodegenerative disease that presents with complex nonmotor symptoms as well as classical motor symptoms. Although medical and surgical treatments alleviate these symptoms, there are no treatments that slow the neurodegenerative process. Eventually, in the late stages of the disease, most patients with PD experience treatment-resistant motor and nonmotor features, such as postural instability, freezing of gait, falling, dysphagia, and cognitive impairment [1]. Therefore, rehabilitation therapies are used as an adjunct treatment to reduce disability and improve the quality of life in patients with PD.

Over the past 20 years, numerous studies investigating the efficacy of rehabilitation therapies in patients with PD have been published [2–8]. These studies have demonstrated the beneficial effect of rehabilitation therapies on motor and swallowing function, activities of daily living, and quality of life. Moreover, recent studies have suggested that intensive rehabilitation might be beneficial in the early stages of PD [9, 10]. Based on this evidence, recent guidelines for the management of PD recommend the referral of patients to physical, occupational, and speech therapists in the early stages of the disease as well as disease-specific rehabilitation therapies [11]. Specific guidelines for physical [12], occupational [13], and speech and swallowing therapies [14] have also been published over the last several years.

Despite this evidence and these guidelines, there is a lack of data addressing the utilization of rehabilitation therapies for patients with PD in practice. Surveys of selected patients with PD have reported that 28% and 60% of patients receive physical therapy (PT) in the Czech Republic [15] and the Netherlands [16], respectively. A study using medical claims data in the Netherlands also reported that 58% of patients with PD received PT over the period of 2013–2015 [17]. Conversely, another claim-based analysis of data collected in the United States demonstrated that only approximately 14% of older patients with PD had claims for rehabilitation therapies during the period of a year [18]. Therefore, the use of rehabilitation therapies in patients with PD may vary in practice, even in developed countries. Moreover, it is unclear whether the present evidence and guidelines for PD have altered clinical practice. There has been no report on the changes of rehabilitation therapy utilization in PD over time.

Therefore, the first aim of this study was to investigate whether the rate of rehabilitation therapy utilization has changed over time in patients with PD, using a nationwide cohort that represented the whole population in Korea. The second aim was to analyze whether the patients' characteristics were associated with rehabilitation therapy utilization in these patients.

## 2. Materials and Methods

### 2.1. Data Source

We used the National Health Insurance Service (NHIS)-National Sample Cohort (NSC) database Version 2.0 for this study [19]. The NHIS is a single, compulsory medical insurance program in South Korea, which was introduced in 1977 and achieved universal coverage by 1989 [19–21]. Therefore, the NHIS contains all of the information regarding health care utilization in Korea. The NHIS-NSC database consisted of 979,390 Korean residents in 2006, equivalent to approximately 2% of the Korean population at that time. The representative nature of this population-based cohort data was ensured through the use of systematic sampling from 2142 strata, based on sex, age group, region of residence, insurance type, and income level. All individuals in the NHIS-NSC database were followed for 14 years (2002–2015) retrospectively and prospectively from 2006. Therefore, the database contains 14 years of all health care utilizations and related information, including diagnoses, procedures, prescription records, demographic information, direct medical costs, and mortality, without any duplications or omissions. Diagnoses were coded according to the International Classification of Diseases, 10th edition. In addition, the database also includes information regarding the registration program for cancer and rare intractable diseases, which was initiated by the Korean government in 2004 to reduce the burden of patients suffering from cancer and 62 rare intractable diseases (e.g., PD) through the use of an additional copayment reduction within the NHI scheme. The NHIS approved the use of the NHIS-NSC database for the present study and provided the database after removing any possibly identifiable information (NHIS-2017-2-607). The study protocol was examined and determined to be exempt from review by the Institutional Review Board of our hospital (E-1712-033-094).

### 2.2. Study Population and Study Periods

We identified patients with PD using the registration code for PD (V124) in the program for rare, intractable disease at 3-year intervals (2004, 2007, 2010, and 2013). Therefore, the study period was divided into 4 periods, each with a 3-year duration: 2004–2006, 2007–2009, 2010–2012, and 2013–2015. For a case to be registered in the program as PD that is eligible for copayment reduction, the physician should confirm the following 3 eligibility criteria in the patient: (1) mild or more severe bradykinesia (a score of at least 2 in the bradykinesia items of the Unified Parkinson's Disease Rating Scale) with at least one of the following features: muscle rigidity, rest tremor, and postural instability, (2) exclusion of secondary parkinsonism, such as stroke, head injury, encephalitis, hypoxic brain injury, and adverse effects of medication, and (3) three or more of the following: unilateral onset, rest tremor, progressive disorder, persistent asymmetry primarily affecting the side of onset, excellent response (70–100%) to levodopa, severe levodopa-induced chorea, levodopa response for 5 years or more, and a clinical course of ten years or more. Although this eligibility criteria is almost the same as that of the UK brain bank criteria [22], the registration program in the NHIS scheme might not definitely exclude atypical parkinsonian syndromes, such as multiple system atrophy, progressive supranuclear palsy, and corticobasal degeneration, which are also rare, intractable diseases. Hence, of these, we excluded individuals with the following diagnoses in each study period: secondary parkinsonism (G21), parkinsonism in diseases that are classified elsewhere (G22), and other degenerative diseases of the basal ganglia (G23). Of these, we also excluded individuals that had no prescription of antiparkinsonian medication within the defined 3-year periods.

### 2.3. Rehabilitation Therapy Utilization

We assessed the utilization of rehabilitation therapies by identifying claims with procedure codes for physical therapy (PT), occupational therapy (OT), and swallowing therapy (ST) in each 3-year study period. We could not assess the utilization of speech therapy, as it is not covered by the NHIS. PT includes complex therapeutic exercise (MM102), isokinetic therapeutic exercise (MM103), rehabilitative development therapy for disorder of the central nervous system (MM105), mattress or mobilization training (MM301), and gait training (MM302). Simple therapeutic exercise (MM101) was excluded from this study because this code represents only 10 minutes of exercise, which is too short for rehabilitation purposes, and is widely used in primary health care service for musculoskeletal problems. OT includes simple occupational therapy (MM111), complex occupational therapy (MM112), special occupational therapy (MM113), and activities of daily living training (MM114). ST includes rehabilitative dysphagia therapy (MX141).

### 2.4. Demographics and Other Characteristics

We identified the demographic data (i.e., sex, age group, income level, and region of residence) and the comorbidities that might require rehabilitation (i.e., cerebrovascular disorders (G46, I60–I63, and I69), traumatic brain injuries (S06.1–S06.6 and S06.8) spinal cord injuries (S14.1, S24.1, and S34.1), multiple sclerosis and other demyelinating disease (G35–G37), central nervous system tumors (C70–C72, D32–33, and D43), and others (G91 and S72)) in each 3-year study period. The number of neurologist and physiatrist visits was also identified from the claims data. The NHIS-NSC database provided the degree of disability (from 1 to 6) for each individual, which was registered in the disability rating system by the Korean government. The disability grade from brain lesion or limb dysfunction was identified from the database. The average number of prescribed daily antiparkinsonian medications and the levodopa-equivalent dose (LED) [23] were calculated based on the claims for drug prescriptions. A list of the antiparkinsonian medications that are mentioned in this study is presented in the Supplementary Table (available here).

### 2.5. Outcomes and Statistical Analysis

The primary outcomes of this study were the utilization of rehabilitation therapies, including PT, OT, and ST in patients with PD. To investigate the effect of patient characteristics on rehabilitation therapy utilization, adjusted odds ratios (AORs) and 95% confidence intervals (CIs) were estimated using multivariable logistic regression analysis during the period of 2013–2015. SAS software version 9.3 (SAS, Inc., Cary, NC, USA) was used for the analyses.

## 3. Results

### 3.1. Characteristics of Study Population

In the NHIS-NSC, the number of patients with PD who met the inclusion criteria was as follows: 384 in 2004, 855 in 2007, 1,023 in 2010, and 1,222 in 2013 (Table 1). The majority of patients were over 60 years of age, and approximately 60% of the patients were female patients, in each group. The numbers of neurologist visits were 6003 (15.63 per person) in 2004–2006, 12546 (14.67 per person) in 2007–2009, 16895 (16.52 per person) in 2010–2012, and 21652 (17.72 per person) in 2013–2015. The numbers of physiatrist visits were 221 (0.58 per person) in 2004–2006, 824 (0.96 per person) in 2007–2009, 2013 (1.97 per person) in 2010–2012, and 3550 (2.91 per person) in 2013–2015.

### 3.2. Rehabilitation Therapy Utilization

**Figure 1:**
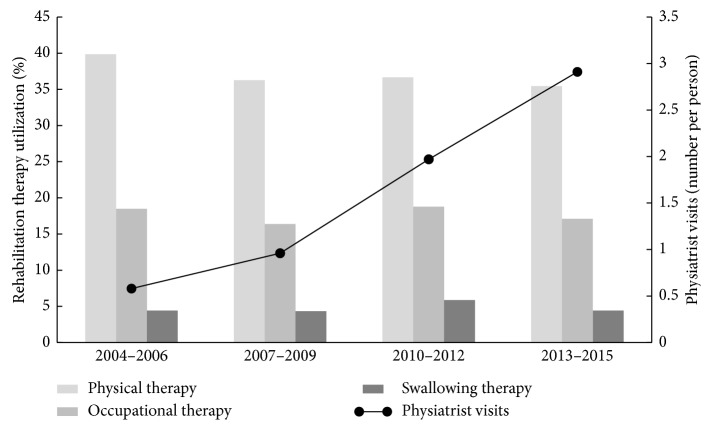
Percentages of rehabilitation therapy utilization and number of physiatrist visits in patients with PD.

Among the patients with PD, 35–40% had claims for PT, 16–19% for OT, and 4–6% for ST. There were no remarkable differences between these rates between the study periods (Figure 1). The number of claims per person for each of the rehabilitation therapies is given in Table 2. While the total number of PT, OT, and ST claims increased gradually throughout the study period, the number of claims per person showed variable results.

### 3.3. Rehabilitation Therapy Utilization According to the Patients' Characteristics

Multivariable logistic regression model analysis of the data from the period of 2013–2015 demonstrated that sex, age, income, disability grade, and LED were significantly associated with rehabilitation therapy utilization in patients with PD (Table 3). Female patients showed higher odds of any rehabilitation therapy (1.668, 95% CI 1.274–2.182) and PT (1.775, 95% CI 1.338–2.301) than the male patients. All of the age groups over 60 years old were more likely to receive any rehabilitation therapy, PT, and OT compared to the age group under 60 years. Patients with PD who were in the age range of 70–79 had the highest odds of receiving rehabilitation therapies (4.053, 95% CI 2.310–7.113 for all types; 4.576, 95% CI 2.557–8.187 for PT; and 3.413, 95% CI 1.635–7.122 for OT) across the age groups. Patients with a high income showed higher odds of receiving any rehabilitation therapy (1.442, 95% CI 1.018–2.045) than those with a low income. Patients who had a disability grade were more likely to receive all types of rehabilitation therapies than patients who did not have a disability grade. Patients with severe disability showed higher odds of receiving therapies than those with mild to moderate disability except OT, for which patients with mild to moderate disability (2.56, 95% CI 1.802–3.635) showed higher odds than those with severe disability (1.8, 95% CI 1.000–3.239). Patients with LED ≥ 800 were more likely to receive all types of rehabilitation therapies than patients with LED < 200.

## 4. Discussion

This is the first study to present the changes in the rate of rehabilitation therapy utilization for over a decade in patients with PD. A number of clinical trials [3, 4, 6, 7] and guidelines [12–14] regarding rehabilitation therapies in PD were published during the investigation period. However, there was no remarkable change in the rate of PT, OT, and ST utilization during this period. In addition, the number of claims for rehabilitation therapies per person did not increase among the patients who were eligible for these claims. These findings may suggest that the evidence and recommendations for the use of rehabilitation therapy in patients with PD have not been adequately integrated in to clinical practice in Korea. Because the number of physiatrist visits increased dramatically during the same period, the lack of rehabilitation facilities and experienced therapists might be one of the causes of the lack of change in the rate of rehabilitation therapy utilization.

Because the appropriate rate of rehabilitation therapy utilization has not been previously defined, it is not clear whether the utilization rates that were recorded in this study were appropriate or not. The utilization rates of PT and OT in this study were higher than those of a previous claim-based study from the United States. This may be due to the fact that the study period of the two studies was different [18]. In addition, the health care system of Korea is quite different from that of the United States as the NHIS covers almost the entire population. All medical facilities, including private institutions, are obliged to provide medical services to NHI subscribers [24]. Conversely, the utilization rate of PT during the 2013–2015 period in this study was around 60% of the rate that was reported in the Netherlands during the same period [17]. Because the Netherlands has developed a specialized care delivery system for patients with PD, the utilization rates in this study do not appear to be optimal. Our study demonstrated that more than 60% and 80% of patients received no PT and OT, respectively, during the 3-year period. Considering the lack of community-based Parkinson's programs in Korea, it could be better for patients with PD to encourage the use of rehabilitation therapy.

The medical costs of the rehabilitation therapies that are required for patients with PD is relatively low in Korea, due to the NHIS and the registration program for rare intractable diseases. Nevertheless, the rate of rehabilitation therapy utilization was significantly lower in low-income patients than in high-income patients. In addition, the utilization of speech therapy was not investigated in this study because it is not covered by the NIHS in Korea. The utilization rate of ST in this study was 4–6%, which is much lower than the utilization rate of speech therapy that has been reported in the United States [18]. The utilization rate of speech therapy is estimated to be very low because patients are required to pay the total cost for speech therapy and because specialized programs for PD, such as the Lee Silverman voice treatment [25], tend to not be available in the majority of clinics in Korea. Therefore, a specialized care delivery system and welfare program for patients with PD is needed to prevent the restriction of the appropriate use of rehabilitation therapies due to medical expenses. Another factor that should be considered is the higher rates of rehabilitation therapy use that were observed in women than in men. One of the causes for this result may be the gender differences in the utilization of health care services in general [26]. The differences in some motor and nonmotor features between men and women with PD may also contribute to the observed difference in the utilization rates [27–29]. These gender differences need to be considered to provide adequate rehabilitation for patients with PD.

The differences in the utilization rates according to age, disability grade, and LED seem to reflect the disease severity of PD. The odds of rehabilitation therapy utilization according to the age groups reached a peak in the 70–79 age range and then slightly decreased above 80 years of age. This may be a natural reduction due to aging and disease progression. Unlike other therapies, OT appeared to be utilized more in patients with mild to moderate disability than those with severe disability. This seems to be a reasonable result reflecting the nature and necessity of OT in the improvement and maintenance of independent activities of daily living.

Because this study is a retrospective analysis of medical claim data, some information might be inaccurate. Fortunately, we were able to identify the cases based on the registration code for rare intractable diseases including PD in this study. The accuracy of the case identification of this study could be verified because the Health Insurance Review and Assessment service in Korea reviews the medical record of each individual at the first registration and verifies the accuracy and reliability of PD diagnosis. However, the information regarding comorbidities, disability grade, and the doses of antiparkinsonian medication may include some errors, such as coding errors, misdiagnosis, and the absence of some medication codes. An additional limitation of this study is that it is difficult to generalize the study results to other countries because the results reflect the specific health care system and socioeconomic situation of Korea. However, the changes over the past decade and the factors that influence the use of rehabilitation therapies may be useful in providing appropriate rehabilitation for patients with PD. In addition, our data will allow comparisons to be made of the status of each country in the rehabilitation of PD.

## 5. Conclusion

This study demonstrated that the rate of rehabilitation therapy utilization did not change remarkably in patients with PD from 2004 to 2015 in Korea, based on the analysis of medical claims data, although the number of physiatrist visits increased dramatically. The present evidence and guidelines may have not been adequately integrated into clinical practice during the period of study. Additional efforts, such as the education of medical professionals, specialized care delivery systems, and welfare programs, may be warranted to provide adequate rehabilitation therapies for patients with PD in clinical practice. The gender differences in these patients should also be considered.

## Figures and Tables

**Table 1:** Characteristics of patients with Parkinson's disease in each 3-year study period.

Characteristics	2004–2006		2007–2009		2010–2012		2013–2015	
	No.	%	No.	%	No.	%	No.	%
Total	384	100	855	100	1023	100	1221	100

*Sex*								
Male	141	37.68	321	37.54	401	39.20	483	39.56
Female	243	62.32	534	62.46	622	60.80	738	60.44

*Age (y)*								
0–59	64	16.67	97	11.35	105	10.26	121	9.91
60–69	159	41.40	232	27.13	249	24.34	276	22.60
70–79	129	33.59	385	45.03	480	46.92	545	44.64
80+	32	8.33	141	16.49	189	18.48	279	22.85

*Income*								
N/A	0	0	95	11.11	77	7.53	74	6.06
Low	92	23.96	171	20.00	193	18.87	253	20.72
Middle	149	38.80	283	33.10	357	34.90	435	35.63
High	143	37.24	306	35.79	396	38.71	459	37.59

*Region of residence*								
Seoul/Incheon	96	25.00	211	24.68	252	24.63	293	24.00
Gyeonggi/Gangwon	84	21.88	187	21.87	240	23.46	314	25.72
Busan/Daegu/Ulsan/Gyeongsang	91	23.70	215	25.15	262	25.61	328	26.86
Daejeon/Sejong/Chungcheong	45	11.72	96	11.23	116	11.34	105	8.60
Gwangju/Jeolla/Jeju	68	17.71	146	17.08	153	14.96	181	14.82

*Comorbidities*								
No	314	81.77	694	81.17	857	83.77	1090	89.27
Yes	70	18.23	161	18.83	166	16.23	131	10.73

*Disability grade*								
None	280	72.92	529	61.87	620	60.61	813	66.58
Mild to moderate (3–6)	59	15.36	189	22.11	249	24.34	323	26.45
Severe (1-2)	45	11.72	137	16.02	154	15.05	85	6.96

*Types of antiparkinsonian medications*								
1-2	219	57.03	531	62.11	632	61.78	764	62.57
3-4	98	25.52	216	25.26	274	26.78	285	23.34
≥5	67	17.45	108	12.63	117	11.44	172	14.09

*Levodopa-equivalent dose*								
<200	69	17.97	165	19.30	195	19.06	234	19.16
≥200, <400	126	32.81	345	40.35	389	38.03	467	38.25
≥400, <600	89	23.18	164	19.18	207	20.23	256	20.97
≥600, <800	46	11.98	70	8.19	105	10.26	118	9.66
≥800	54	14.06	111	12.98	127	12.41	146	11.96

*Number of visits*								
Neurologists	6003	—	12546	—	16895	—	21652	—
Physiatrists	221	—	824	—	2013	—	3550	—

**Table 2:** Number of claims for each rehabilitation therapies.

	Types of rehabilitation therapies	2004–2006	2007–2009	2010–2012	2013–2015
PT	Complex therapeutic exercise (MM102)	442	747	1226	1380
	Isokinetic therapeutic exercise (MM103)	0	10	66	146
	Rehabilitative development therapy for disorder of the central nervous system (MM105)	656	1068	2396	3021
	Mattress or mobilization training (MM301)	394	724	1298	1502
	Gait training (MM302)	705	1075	1795	2639
	Total	2197	3624	6781	8688
	Per person	14.36	11.69	18.08	20.06

OT	Simple occupational therapy (MM111)	18	60	142	51
	Complex occupational therapy (MM112)	1138	1299	2139	2582
	Special occupational therapy (MM113)	112	427	756	1462
	Activities of daily living training (MM114)	212	362	832	980
	Total	1480	2148	3869	5075
	Per person	20.85	15.34	20.15	24.28

ST	Rehabilitative dysphagia therapy (MX141)	374	450	766	792
	Per person	22	12.16	12.77	14.67

Simple therapeutic exercise (MM101) was excluded in this study because this code serves only 10 minutes of exercise, which is too short for the rehabilitation purpose, and widely used in primary health care service for musculoskeletal problems. PT, physical therapy; OT, occupational therapy; ST, swallowing therapy.

**Table 3:** Rehabilitation therapy use according to patients' characteristics in the period of 2013–2015.

Characteristics	All types		PT		OT		ST	
	AOR	95% CI	AOR	95% CI	AOR	95% CI	AOR	95% CI
*Sex*								
Male	Ref.		Ref.		Ref.		Ref.	
Female	1.668	1.274–2.182	1.755	1.338–2.301	1.214	0.868–1.698	0.894	0.493–1.619

*Age (y)*								
0–59	Ref.		Ref.		Ref.		Ref.	
60–69	2.806	1.550–5.077	3.139	1.703–5.785	2.483	1.144–5.391	4.13	0.872–19.550
70–79	4.053	2.310–7.113	4.576	2.557–8.187	3.413	1.635–7.122	3.566	0.782–16.260
80+	3.773	2.068–6.884	4.041	2.172–7.516	3.246	1.475–7.142	2.99	0.585–15.293

*Income*								
Low	Ref.		Ref.		Ref.		Ref.	
Middle	1.095	0.767–1.565	1.125	0.786–1.610	0.965	0.614–1.516	1.033	0.411–2.598
High	1.442	1.018–2.045	1.412	0.994–2.006	1.219	0.791–1.879	2.066	0.902–4.736

*Region of residence*								
Seoul/Incheon	Ref.		Ref.		Ref.		Ref.	
Gyeonggi/Gangwon	0.825	0.571–1.192	0.852	0.590–1.237	1.106	0.696–1.757	0.887	0.411–1.913
Busan/Daegu/Ulsan/Gyeongsang	0.921	0.639–1.327	0.954	0.661–1.377	1.27	0.807–1.999	0.465	0.189–1.145
Daejeon/Sejong/Chungcheong	0.879	0.535–1.445	0.904	0.548–1.489	1.034	0.546–1.957	1.117	0.404–3.090
Gwangju/Jeolla/Jeju	0.806	0.525–1.239	0.871	0.566–1.340	0.952	0.546–1.661	0.814	0.315–2.106

*Comorbidities*								
No	Ref.		Ref.		Ref.		Ref.	
Yes	1.457	0.976–2.175	1.397	0.935–2.087	1.546	0.977–2.446	1.758	0.821–3.767

*Disability grade*								
None	Ref.		Ref.		Ref.		Ref.	
Mild to moderate (3–6)	2	1.486–2.690	2.079	1.544–2.800	2.56	1.802–3.635	2.453	1.295–4.646
Severe (1-2)	2.939	1.815–4.758	2.738	1.692–4.430	1.8	1.000–3.239	3.078	1.214–7.802

*Levodopa-equivalent dose*								
<200	Ref.		Ref.		Ref.		Ref.	
≥200, <400	1.016	0.703–1.470	1.076	0.741–0.903	0.951	0.581–1.557	2.284	0.638–8.176
≥400, <600	1.338	0.882–2.030	1.376	0.903–2.096	1.234	0.715–2.129	3.103	0.828–11.625
≥600, <800	1.647	0.994–2.730	1.768	1.064–2.940	1.344	0.706–2.560	2.964	0.695–12.642
≥800	4.05	2.484–6.601	3.952	2.423–6.446	3.792	2.174–6.616	9.438	2.601–34.253

PT, physical therapy; OT, occupational therapy; ST, swallowing therapy.

## Data Availability

The National Health Insurance Sharing Service provides the original data used in this study (https://nhiss.nhis.or.kr/bd/ab/bdaba011eng.do). The data used to support the findings of this study are available from the corresponding authors upon request.

## Supplementary Materials

Supplementary Materials Supplementary Table: list of antiparkinsonian medications mentioned in the present study. Classification, generic name, and generic code are presented.
