# Supplementary material for: Rehabilitation Therapy Utilization in Patients with Parkinson's Disease in Korea
Source: Parkinsons Dis. 2018 Nov 14;2018:9475415. doi: 10.1155/2018/9475415 (PMC6261078; doi:10.1155/2018/9475415)
Supplement: Supplementary Materials — Supplementary Table: list of antiparkinsonian medications mentioned in the present study. Classification, generic name, and generic code are presented. [file 9475415.f1.docx]

Supplementary Table. List of antiparkinsonian medications mentioned the present study.

| Classification | Medication | Generic code |
| --- | --- | --- |
| Levodopa | Levodopa | 183001ATB, 256000ACH, 256000ATB, 256000ATD, 256000ATR, 256100ATR, 256200ATB, 256300ATB, 256400ATB |
|  | Controlled release levodopa | 255900ACR, 256000ACR, 256500ATR |
|  | Duodopa | 639400ACM |
| COMT inhibitors | Entacapone | 439201ATB |
|  | Levodopa-Entacapone | 468300ATB, 468400ATB, 468900ATB, 499900ATB, 507200ATB, 507300ATB |
| Dopamine agonists | Pramipexole | 402501ATB, 402502ATB, 402503ATB, 402504ATB, 402505ATB, 402505ATR, 402506ATR, 402507ATR |
|  | Ropinirole | 224901ATB, 224902ATB, 224903ATB, 224904ATB, 224902ATR, 224905ATR, 224906ATR |
|  | Rotigotine | 612107CPC, 612108CPC, 612109CPC, 612110CPC, 612111CPC, 612112CPC |
|  | Piribedil | 213901ATB |
|  | Lisuride | 184601ATB |
|  | Bromocryptine | 118701ATB |
|  | Pergolide | 211201ATB, 211202ATB, 211203ATB |
| MAOB inhibitors | Selegiline | 226401ATB |
|  | Rasagiline | 625201ATB |
| Other | Amantadine | 106101ACH, 106201ATB |

COMT, catechol-O-methyl transferase; MAOB, monoamine oxidase type B
